# Properties of Cellulose Pulp and Polyurethane Composite Films Fabricated with Curcumin by Using NMMO Ionic Liquid

**DOI:** 10.3390/gels8040248

**Published:** 2022-04-18

**Authors:** Chaehyun Jo, Sam Soo Kim, Balasubramanian Rukmanikrishnan, Srinivasan Ramalingam, Prabakaran D. S., Jaewoong Lee

**Affiliations:** 1 Department of Fiber System Engineering, Yeungnam University, 280 Daehak-Ro, Gyeongsan 38541, Korea; jo1114@ynu.ac.kr (C.J.); sskim@yu.ac.kr (S.S.K.); 2 Department of Food Science and Technology, Yeungnam University, 280 Daehak-Ro, Gyeongsan 38541, Korea; sribt27@gmail.com; 3 Department of Radiation Oncology, College of Medicine, Chungbuk National University, Chungdae-ro 1, Seowon-gu, Cheongju 28644, Korea; prababio@gmail.com; 4 Department of Biotechnology, Ayya Nadar Janaki Ammal College (Autonomous), Srivilliputhur Main Road, Sivakasi 626124, Tamil Nadu, India

**Keywords:** cellulose pulp, polyurethane, curcumin, antioxidant properties, non-cytotoxicity, barrier properties, mechanical properties

## Abstract

Cellulose pulp (CP), polyurethane (PU), and curcumin-based biocompatible composite films were prepared using a simple cost-effective method. Significant structural and microstructural changes were studied using FT-IR spectroscopy, XRD, and SEM. The 5% and 10% gravimetric losses of the CP/PU/curcumin composite were found to be in the range 87.2–182.3 °C and 166.7–249.8 °C, respectively. All the composites exhibited single T_g_ values in the range 147.4–154.2 °C. The tensile strength of CP was measured to be 93.2 MPa, which dropped to 14.1 MPa for the 1:0.5 CP/PU composite and then steadily increased to 30.5 MPa with further addition of PU. The elongation at the break of the composites decreased from 8.1 to 3.7% with the addition of PU. The addition of PU also improved the water vapor permeability (3.96 × 10^−9^ to 1.75 × 10^−9^ g m^−1^ s^−1^ Pa^−1^) and swelling ratio (285 to 202%) of the CP composite films. The CP/PU/curcumin composite exhibited good antioxidant activity and no cytotoxicity when tested on the HaCat cell line. The visual appearance and UV transmittance (86.2–32.9% at 600 nm) of the CP composite films were significantly altered by the incorporation of PU and curcumin. This study demonstrates that CP/PU/curcumin composites can be used for various packaging and biomedical applications.

## 1. Introduction

In recent years, sustainable and environmentally friendly materials have garnered significant attention in both scientific and industrial research fields. This is because they are abundant; low-cost; present low risk to animals, humans, and other living organisms; carbon neutral, biodegradable and biocompatible [1,2,3,4,5]. These concerns and challenges have led to the development of novel environment-friendly and biodegradable packaging materials. Among natural biopolymer resources, biomass has evolved as a value-added product for various industrial applications. Biomass-based nanocomposites are novel composites consisting of biodegradable biopolymers and bio-nanofillers. Recently, several researchers fabricated metal nanocomposites using different plant-based and natural biopolymers and revealed the least cytotoxicity and higher antimicrobial properties. Cellulose pulp (CP) is the most abundant, renewable, and inexpensive lignocellulosic material consisting of D-glucopyranose units, which is widely used in the paper and agricultural industries [6,7,8,9]. However, the brittleness and solubility of cellulose pulp restrict its application. Owing to its partly crystalline and partly amorphous structure, cellulose pulp is poorly soluble in many organic solvents [10,11,12,13,14,15,16,17]. A combination of 10% NaOH, NaOH/urea, and DMA/LiCl can dissolve cellulose pulp, but it requires a relatively lengthy pre-treatment process. Ionic liquids are promising green solvents for dissolving cellulose pulp and are rapidly replacing organic solvents. Moreover, they have been used to prepare various composites by blending cellulose, wood pulp, and chitosan [18,19].

The addition of organic polymers, inorganic nanoparticles, and natural fillers to polymer backbones has gained popularity because they help improve the thermal, mechanical, and water barrier properties of composites. For instance, polyurethane (PU) is a block copolymer consisting of both soft and hard segments. PU-based composites have been extensively studied and widely used in polymer and materials sciences owing to their excellent mechanical, gas barrier, and water barrier properties [20,21,22,23,24,25]. They have several biomedical applications, such as in the field of tissue engineering; they also have important medical and industrial applications because of their exceptional biocompatibility and biodegradability. A synergistic combination of cellulose pulp and PU has been shown to reduce the dependence on synthetic resources to a certain degree; in addition, such plant-based natural fiber-reinforced polymer matrix composites promote the use of renewable resources [26,27,28,29,30].

Turmeric (Curcuma longa) is a rhizomatous perennial plant. It is a rich source of phenolic compounds, such as curcuminoids. Curcumin (1,7-bis(4-hydroxy-3-methoxyphenyl)-1,6-heptadiene-3,5-dione) is a natural bioactive compound extracted from turmeric powder. It has a low molecular weight and a phenolic structure consisting of α,β-unsaturated carbonyl groups. Curcumin has various therapeutic applications because of its antimicrobial, antioxidant, and anti-inflammatory properties. Moreover, it is compatible with different types of polymers [31,32,33,34,35,36]. We hypothesize that the incorporation of PU and curcumin into CP can improve the antioxidant, mechanical, and thermal properties of the resulting composite films.

To test our hypothesis, in this study, we prepared composites based on a synergistic novel combination of CP, PU, and curcumin by simple dissolution in N-methylmorpholine N-oxide (NMMO) solvent. We thoroughly investigated the thermal, mechanical, and water barrier properties of these sustainable composites by performing Fourier transform infrared (FT-IR) spectroscopy, X-ray diffraction (XRD), scanning electron microscopy (SEM), and UV spectroscopy. In addition, we examined the antioxidant activity and cytotoxicity of these CP/PU/curcumin composites. In this study, we highlight the properties that make these composites suitable for packaging applications.

## 2. Experimental Section

### 2.1. Materials

Cellulose pulp was purchased from Moorim P & P Co., Ltd., (Ulsan, South Korea) and curcumin was purchased from Sigma-Aldrich (Yongin, South Korea). NMMO and curcumin powder were purchased from Dajang Chemicals (Siheung, South Korea). Polyurethane (Elastollan^®^ 1283 D11 U) was purchased from BASF (Seoul, South Korea). Water-soluble tetrazolium salt (WST-1) cat. #EZ-1000 cytox was purchased from Daeil Lab Service Co. (Seoul, South Korea), to measure cytotoxicity. Note that all the reagents were used as received without further purification.

### 2.2. Preparation of CP/PU/Curcumin Composite Films

First, CP (4 g), PU (4 g), and curcumin (10 wt%) were mixed thoroughly using a hand blender. Next, the CP/PU/curcumin mixture was dispersed in a round-bottom (RB) flask containing NMMO (92 g) and heated to 100 ºC. The RB flask was tightly closed and dipped in an oil bath to maintain uniform temperature and avoid recrystallization of the NMMO solvent at the top of the flask. After 2.3 h, the RB flask was removed and kept in a vacuum oven at 100 ºC for another 6 h to completely remove air bubbles. Subsequently, a viscous CP/PU/curcumin solution at 80 °C was spread uniformly on a glass surface. The glass plate was immediately transferred to a coagulation bath containing water, where it was kept for 6 h and fresh water was transferred every 1 h without disturbing the film. Following the complete removal of the solvent from the CP/PU/curcumin composite film, it was transferred to another glass plate and dried at room temperature for 48 h. The thickness of the resulting transparent films was in the range of 0.07–0.10 mm. The prepared composite films were stored at 30 °C and 60 ± RH. All CP/PU/Curcumin composites were prepared using the above-mentioned procedure. A series of composite films were prepared using a similar method, such that they consisted of only CP; 1:0.5 CP/PU; 1:1 CP/PU and 1:1 CP/PU with 10 wt% curcumin (Table S1). The characterization methods of these composite films are provided in the Supporting Information

## 3. Results and Discussion

### 3.1. FT-IR Spectroscopy

**Figure 1:**
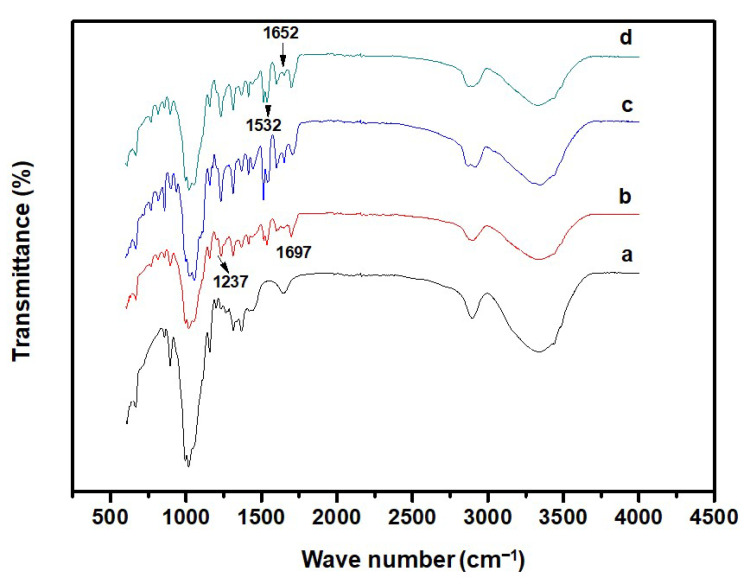
FT−IR spectra of composite films having: (**a**) only CP, (**b**) 1:0.5 CP/PU, (**c**) 1:1 CP/PU, and (**d**) 1:1 CP/PU with 10 wt% curcumin.

The FT-IR analysis of the CP/PU/curcumin blend films was carried out to study the interaction between the polymeric materials, and the resulting FT-IR spectra were shown in (Figure 1). The broadband observed at 3100–3450 cm^−1^ (for both CP and CP/PU composites) corresponds to the stretching vibration of the O–H bond. The peaks at 2893 cm^−1^ and 1367 cm^−1^ correspond to the stretching vibrations of the C–H bond and bending vibrations of the O–H bond in cellulose, respectively. The peak at 858 cm^−1^ results from the vibrations of the C–H bond, which is commonly observed in neat cellulose pulp [37,38]. The band at 895 cm^−1^ can be attributed to the stretching vibrations of C–O–C in the β-(1-4)-glycosidic linkages of cellulose, which is considered to be in the amorphous phase. PU added CP composites showed characteristic bands at 1697 cm^−1^, 1313 cm^−1^, and 1231 cm^−1^ owing to the stretching vibrations of C=O, C–N, and C–O in the urethane group, respectively [39]. It also exhibited bands corresponding to the carbonyl (1650–1850 cm^−1^), amide (1420–1650 cm^−1^) and ether (910–1250 cm^−1^) groups. The characteristic peaks of curcumin at 1652 cm^−1^, 1532 cm^−1^, and 1446 cm^−1^ correspond to the vibrations of the carbonyl group C=O, stretching vibrations of the C–C bond in the benzene ring, and olefinic bending vibrations of the C–H bond in the benzene ring exhibited in 1:1 CP/PU with 10 wt% curcumin composites, respectively. The intensity and positions of the peaks in the 1:1 CP/PU composite spectrum were observed to change when 10 wt% curcumin was added, indicating that the CP, PU, and curcumin bonded with each other in the CP/PU/curcumin composite. Characteristic peaks at 1357 cm^−1^ (C–O–H bending), 1017 cm^−1^ (C–O stretching), and 1704 cm^−1^ (PU-carbonyl group) shifted to lower wavenumbers, namely, 1372, 1056, and 1697 cm^−1^, respectively. These results suggest that hydrogen bond interactions were present in the CP/PU/curcumin composite films.

### 3.2. XRD Analysis

**Figure 2:**
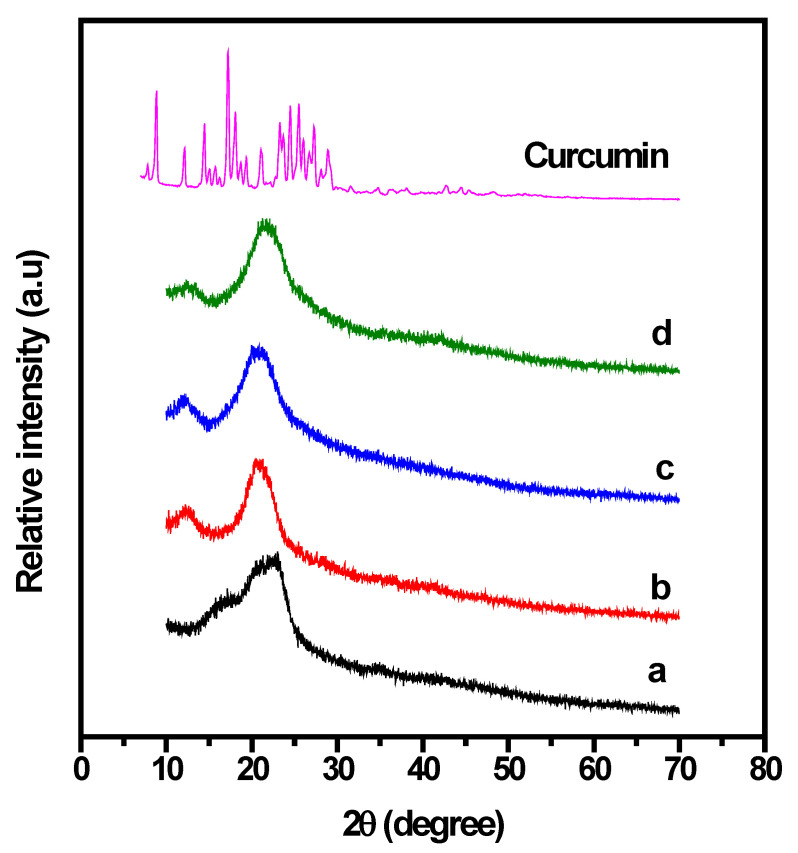
XRD patterns of composite films having: (**a**) only CP, (**b**) 1:0.5 CP/PU, (**c**) 1:1 CP/PU, and (**d**) 1:1 CP/PU with 10 wt% curcumin.

The miscibility and compatibility of the materials of each film were studied by XRD pattern. The reinforcement effect of PU on CP was characterized using XRD analysis, and the corresponding XRD patterns are shown in Figure 2. It is well known that the width and intensity of the peaks in an XRD pattern are related to the crystalline structure of the underling material. The XRD pattern of neat CP exhibited a peak of about 2θ = 16–23°, which indicates a crystalline phase. However, after the dissolution of PU, the structure of CP changed, and the diffraction peaks of the 1:0.5 CP/PU and 1:1 CP/PU composites were observed to shift. The amorphous nature of PU significantly altered the intensity of the XRD peaks and a broad peak was exhibited by the 1:1 CP/PU composite with 10 wt% curcumin. The crystallinity of the CP/PU composites gradually decreased owing to the dissolution of PU in the CP matrix. Broader peaks indicate an increase in the amorphous phase of the CP composites, which in turn increases the solubility of the composites in the NMMO solvent and helps form intermolecular hydrogen bonds between the components. The addition of PU to CP increased its amorphous nature such that the XRD peaks of the CP/PU composites were relatively broad with high intensity. Furthermore, peak shifts were observed in the XRD patterns of the CP/PU and CP/PU/curcumin composite films. The XRD pattern of curcumin showed intense diffraction peaks between 10° and 30° owing to its highly crystalline structure. The substantial decrease in the peak intensity of the CP/PU/curcumin composite clearly indicates the interaction between all the components in the polymer backbone. The absence of peaks corresponding to curcumin in the XRD pattern of 1:1 CP/PU composite with 10 wt% curcumin indicates that curcumin is properly combined with the CP/PU biopolymer matrix and is present in a low-order or amorphous state. These results confirm the proper dispersion of materials in the solvent and are in good agreement with the results of previous studies [6,35,40,41,42,43].

### 3.3. UV Spectroscopy

**Figure 3:**
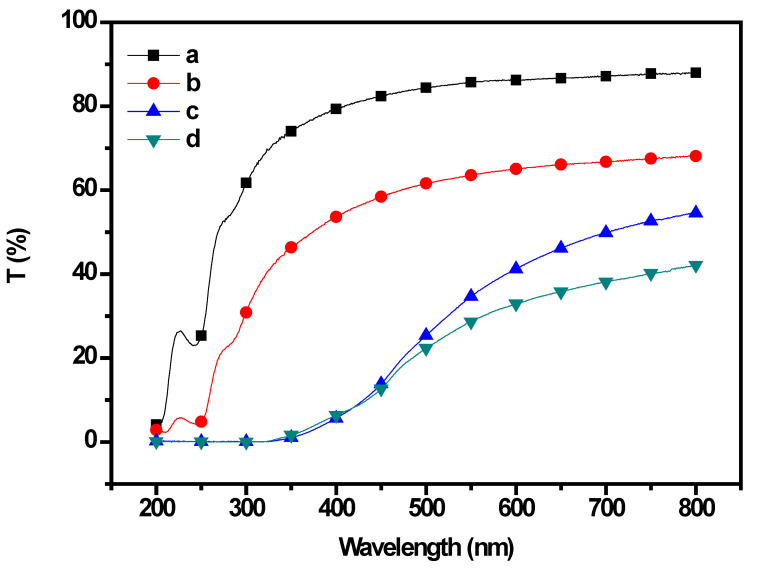
UV transmittance spectra of composite films having: (**a**) only CP, (**b**) 1:0.5 CP/PU, (**c**) 1:1 CP/PU, and (**d**) 1:1 CP/PU with 10 wt% curcumin.

UV light protection is one of the properties in great demand in multifunctional materials. The thickness of the CP/PU/curcumin composite films was in the range of 0.07–0.1. The surface color and visual appearance of the CP films were affected owing to the addition of PU. The neat CP films appeared translucent, whereas the 1:0.5 CP/PU, 1:1 CP/PU, and 1:1 CP/PU with 10 wt% curcumin films appeared slightly opaque. The Hunter L* (lightness) value of the composites decreased, whereas the Hunter a* (redness) and b* (yellowness) values increased with increasing PU content. The neat CP composite film was colorless and transparent; however, the composite films containing PU and curcumin exhibited a yellow color. The UV transmittance of the CP/PU/curcumin composites as a function of wavelength is shown in Figure 3 (also, see Table 1). The UV transmittance of neat CP was 86.2% at 600 mm; however, it decreased significantly after the incorporation of PU. The transmittance of 1:0.5 CP/PU, 1:1 CP/PU, and 1:1 CP/PU with 10 wt% curcumin was 65.1, 41.2, and 32.9%, respectively. Note that the 1:1 CP/PU composite and 1:1 CP/PU composite with 10 wt% curcumin almost completely blocked the UV light below 400 mm. This indicates that the materials were well dispersed in the solvent and the resulting composites are suitable for packaging applications.

### 3.4. Water Vapor Permeability

The water vapor permeability (WVP) is the most important ertyin food packaging application to control the moisture transfer between the food and the surrounding atmosphere. The WVP of the CP/PU/curcumin composites was measured to determine their water barrier properties, and the corresponding data are summarized in Table 1. The WVP of neat CP was measured to be 3.96 × 10^−9^ g m^−1^ s^−1^ Pa^−1^, which decreased to 2.34 × 10^−9^ g m^−1^ s^−1^ Pa^−1^ on the addition of PU. Note that both PU and curcumin are hydrophobic in nature; moreover, when PU and curcumin are dispersed throughout the CP matrix, a tortuous path for the diffusion of water molecules is created that increases the diffusion path length. The WVP of the 1:1 CP/PU composite was 1.81 × 10^−9^ g m^−1^ s^−1^ Pa^−1^, which decreased to 1.75 × 10^−9^ g m^−1^ s^−1^ Pa^−1^ with the addition of curcumin powder. Note, that the reduced hydrophilicity of the CP/PU/curcumin composites makes them suitable for packaging applications.

### 3.5. Swelling Ratio

The swelling behavior of the CP/PU/curcumin composites was studied in water at 25 °C. A low swelling ability is attributed to a high cross-link density of the composite films. This is because, at higher cross-link densities, water permeation into the matrix becomes more difficult, which in turn influences the swelling capacity of the gel. The SR of neat CP decreased from 285% to 262% with the addition of PU. Thus, the SR of neat CP was 32% higher than that of the 1:1 CP/PU composite. The SR further decreased to 202% for the CP/PU-1:1 composite with 10 wt% curcumin. Therefore, the hydrophobic nature of PU and curcumin fillers hinders the diffusion of the water molecules through the composite films and significantly influences the swelling properties of CP.

### 3.6. Contact Angle

The surface hydrophilicity/hydrophobicity of the composite films is an important determining factor for packaging applications. It is determined by measuring the water CA, whose values are listed in Table 1. The results indicate that the addition of PU increased the hydrophobicity of the composite films. The CA of neat CP was 51.4°, which increased to 55.6° and 58.2° for the 1:0.5 CP/PU and 1:1 CP/PU composites, respectively. Furthermore, the addition of curcumin slightly increased the CA of the 1:1 CP/PU composite film. This is owing to the hydrophobic nature of the curcumin fillers, which hinder the diffusion of water molecules through the composite films. This result is consistent with the SR values of the CP/PU/curcumin composites.

### 3.7. Thermogravimetric Analysis

**Figure 4:**
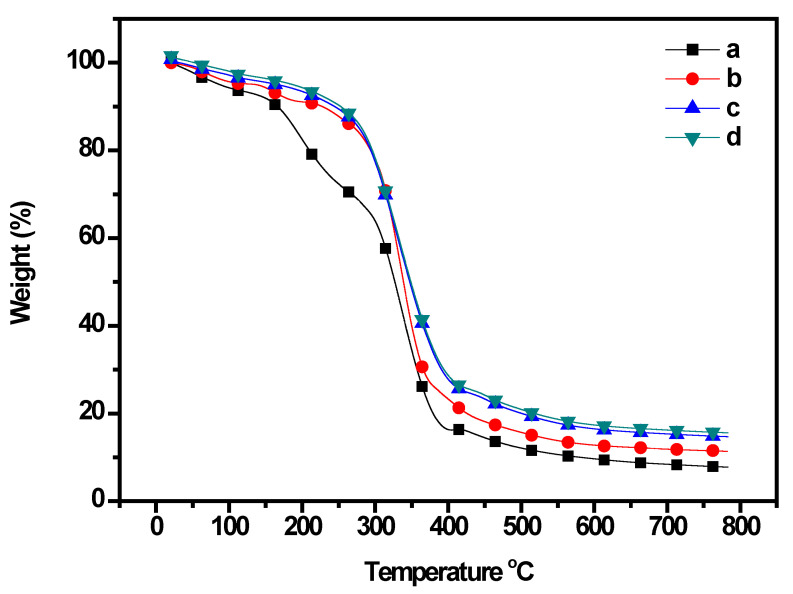
TGA curves of composites having: (**a**) only CP, (**b**) 1:0.5 CP/PU, (**c**) 1:1 CP/PU, and (**d**) 1:1 CP/PU with 10 wt% curcumin.

The characteristic thermal stability of the CP/PU/curcumin composites was studied using TGA in an O_2_ atmosphere and the results are presented in Figure 4 and Table 2. All composites exhibited three stages of weight loss. PU significantly improved the thermal stability of CP. The 5% and 10% gravimetric losses of the composites were in the range 87.2–182.3 °C and 166.7–249.8 °C, respectively. The initial degradation temperature of neat CP was 87.2 °C, which increased to 136.8, 172.1, and 182.3 °C for the 1:0.5 CP/PU, 1:1 CP/PU, and 1:1 CP/PU with 10 wt% curcumin composites, respectively. We found that adding PU to the CP matrix caused a shift in the first and third degradation temperatures towards higher temperatures (approximately 130–230 °C and 250–450 °C, respectively). However, the weight loss was nearly constant during the second degradation stage. The char yield of neat CP was 7.8%, which increased to 15.7% for the 1:1 CP/PU with 10 wt% curcumin composites. The improvement in the thermal stability of the CP/PU/curcumin composite can be ascribed to a confined network structure and uniform dispersion of the components in the polymer matrix. Furthermore, the incorporation of PU leads to synergistic interfacial interactions, which slows down the thermal decomposition of these composite films [28,39].

### 3.8. Differential Scanning Calorimetry

**Figure 5:**
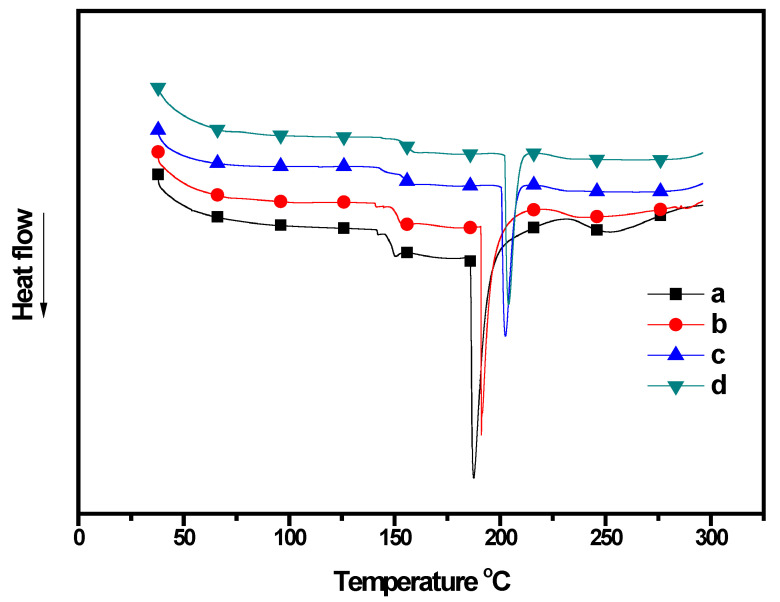
DSC thermogram of composites having: (**a**) only CP, (**b**) 1:0.5 CP/PU, (**c**) 1:1 CP/PU, and (**d**) 1:1 CP/PU with 10 wt% curcumin.

The DSC thermogram revealed T_g_ and T_m_ of the CP/PU/curcumin composites and the results are summarized in Table 2 and Figure 5. The addition of PU seemed to improve T_g_ and T_m_ of neat CP. The 1:1 CP/PU with 10 wt% curcumin composite exhibited the highest T_g_ (154.2 °C) and T_m_ (204.2 °C) values, which is owing to the higher order interactions and relaxation mechanisms in this composite. All composite films exhibited single T_g_ and T_m_ peaks. However, the intensity of the T_m_ peaks decreased with the addition of PU to the composites. The significant improvement in the T_g_ and T_m_ values indicates the restricted molecular motion in the composite network, which is formed by the covalent cross-linking and strong hydrogen bonding between the CP and PU networks. These results are consistent with that of the XRD analysis and the mechanical properties of the composites [26,39,44,45].

### 3.9. Mechanical Properties

The mechanical properties of the hydrogel films are important for the food packaging application during shipping, handling and storage. The mechanical properties of the CP/PU/curcumin composites are listed in Table 2. The tensile strength (TS) of neat CP was 93.2 MPa; however, it decreased to 14.1 MPa in the 1:0.5 CP/PU composite. The TS increased again to 29.5 MPa in the 1:1 CP/PU composite. The sudden decrease in TS was due to the dilution effect, poor adhesion between the polymer matrices leads to the formation of numerous voids at the matrix interface. The elongation at break (EB) of the CP was 8.1% which decreased to 3.7 and 3.9 with the addition of polyurethane. This decrease in EB on the addition of PU can be attributed to a decrease in polymer extensibility, which is caused by the formation of microphase separations in the composite films. These results are consistent with those of the SEM analysis, which revealed the CP/PU/curcumin composites to have an uneven and rough surface that may be responsible for the low EB [46]. Note that a low aspect ratio and particulate nature can also lead to a low EB [47]. Overall, the CP/PU/curcumin composites were found to be hard and brittle in nature.

### 3.10. SEM Analysis

**Figure 6:**
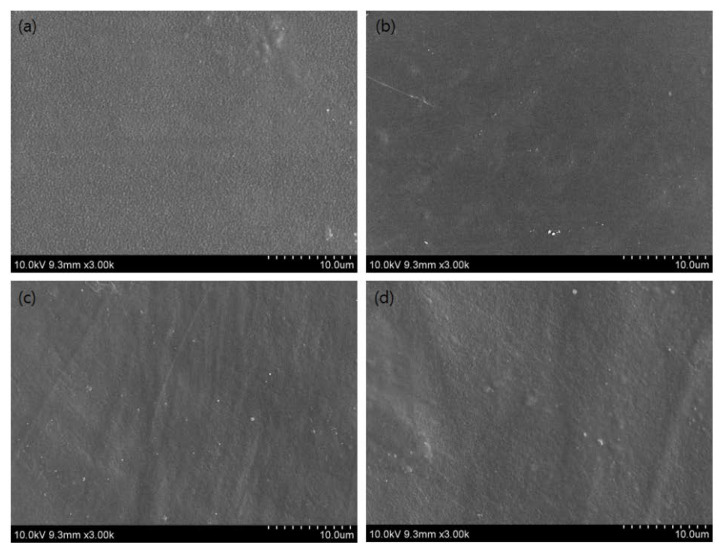
SEM images of composites having: (**a**) only CP, (**b**) 1:0.5 CP/PU, (**c)** 1:1 CP/PU, and (**d**) 1:1 CP/PU with 10 wt% curcumin.

**Figure 7:**
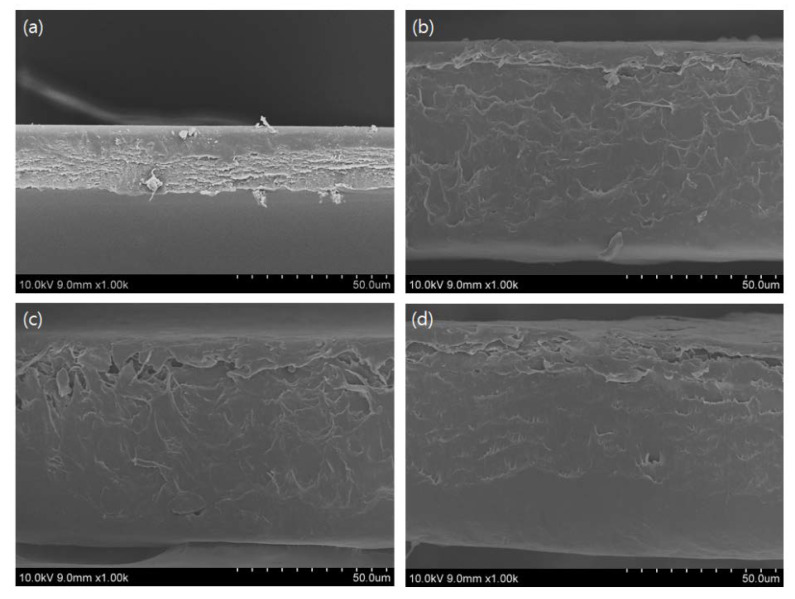
Cross-sectional SEM images of composites having: (**a**) only CP, (**b**) 1:0.5 CP/PU (**c**) 1:1 CP/PU, and (**d**) 1:1 CP/PU with 10 wt% curcumin.

Scanning electron microscopy (SEM) analysis was carried out to characterize the microstructure morphology and homogeneity of the CP/PU/Curcumin composite films with different ratios. Figure 6 and Figure 7 show the surface and cross-sectional SEM images of the composite films, respectively. The neat CP film had a homogenous surface without any agglomerates. However, the 1:0.5 CP/PU and 1:1 CP/PU composites had less homogenous surfaces; in addition, they exhibited small, uneven, and discontinuous voids. The cross-sectional image of the neat CP film shows that it has a slightly rough and uneven surface. The addition of PU increased the surface roughness of the composites. Note, that these results are consistent with the mechanical properties of the composite films. The voids and cavities revealed by the cross-sectional SEM images are the main cause of the low TS and EB of the CP/PU/curcumin composite films. These results suggest that cellulose pulp, polyurethane and curcumin based composite films possess reasonably good mechanical properties. The SEM results were consistent with the mechanical and water absorption properties of the composite films.

### 3.11. Antioxidant Activity

**Figure 8:**
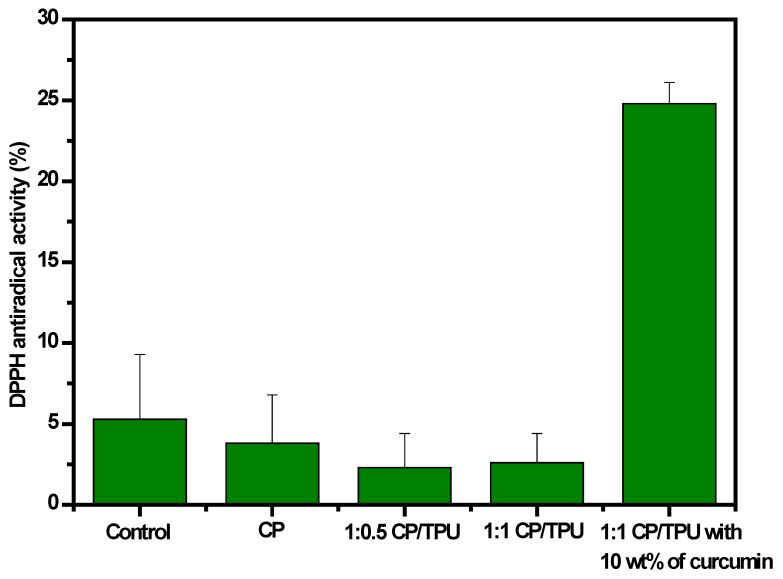
DPPH radical scavenging activity of composites.

Packaging films that have antioxidant properties are extremely effective in protecting food materials. The antioxidant activity of the CP/PU/curcumin composite films was determined using the DPPH radical scavenging activity (see Figure 8). As expected, the neat CP, 1:0.5 CP/PU, and 1:1 CP/PU composites showed no antioxidant activity. Curcumin is well known for its strong antioxidant activity, as it acts as a superoxide radical scavenger and singlet oxygen quencher. The 1:1 CP/PU with 10 wt% curcumin composite showed good antioxidant activity (24.8%) after an incubation period of 1 h. Curcumin is reported to have good thermal stability, as heating does not affect its active compounds, such as curcumin and phenolic hydroxyl groups. Some studies have pointed out that the β-diketone moiety plays an important role in the antioxidant mechanism of curcumin; for instance, the donation of an H-atom from the β-diketone moiety to a lipid alkyl or a lipid peroxyl radical has been shown to contribute to the antioxidant activity of curcumin. The high antioxidant activity of the CP/PU/curcumin composites makes them potential candidates for antioxidant food packaging material.

### 3.12. Cytotoxicity

**Figure 9:**
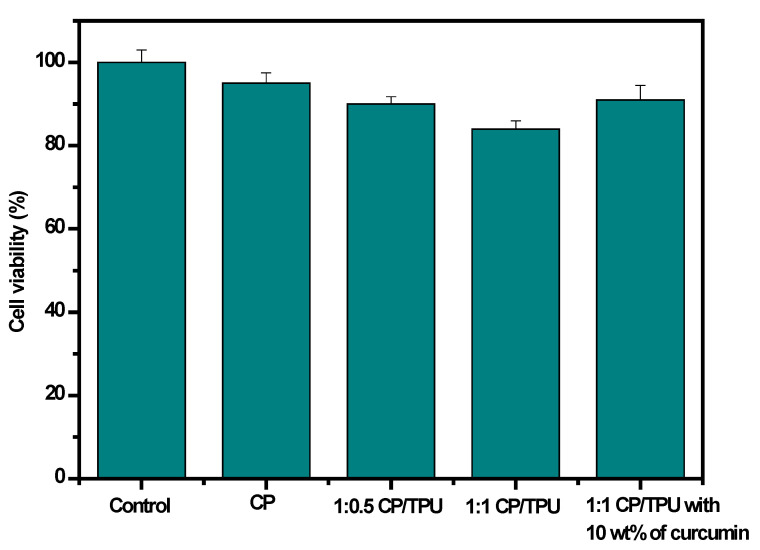
Cell viability of HaCaT cell line for composites.

**Figure 10:**
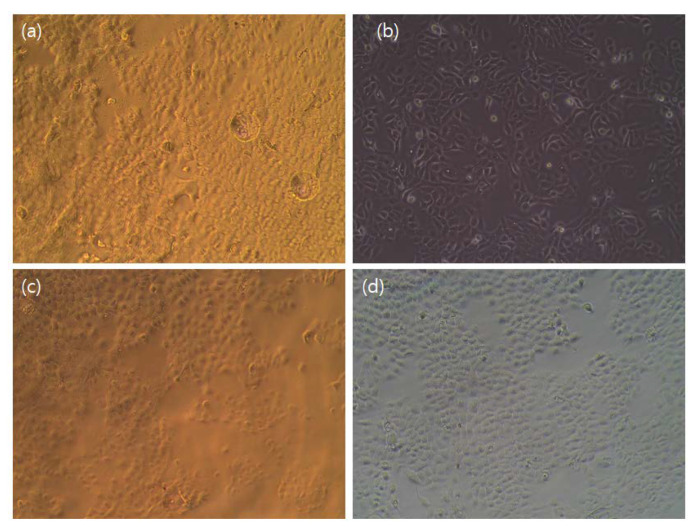
Light microscopy images of HaCaT cell line for composites having: (**a**) only CP, (**b**) 1:0.5 CP/PU, (**c**) 1:1 CP/PU, and (**d**) 1:1 CP/PU with 10 wt% curcumin.

Cell viability is an essential parameter to test the biosafety of a material. The cytotoxicity of the synthesized CP, CP/PU, and CP/PU/curcumin composites was evaluated using the WST-1 assay method on human keratinocyte cells (i.e., the HaCaT cell line), and the results are summarized in Figure 9 and Figure 10. All the different composites were found to be non-cytotoxic. There was no difference in cell viability between the control and neat CP films. However, cell viability decreased in the 1:0.5 CP/PU and 1:1 CP/PU composites. Note that the cell viability of the 1:1 CP/PU with 10 wt% curcumin composite was better than that of the other CP/PU composites. The decrease in cell viability was caused by cellular stress owing to the presence of a foreign element in the cell medium. The cell viability of the 1:1 CP/PU with 10 wt% curcumin composite was 91%, which can be attributed to the disappearance of cellular growth that is favorable for cell proliferation. This result is in good agreement with those of previous studies [35,48]. The above results show that the CP/PU/curcumin composites are biocompatible and safe to be used for packaging applications.

## 4. Conclusions

In this study, we successfully blended two sustainable materials, namely, CP and curcumin, with a commercially available synthetic material, namely, PU, using an ionic solvent, namely, NMMO. The apparent color and UV transmittance of the composite films were significantly changed owing to the addition of PU. XRD analysis showed that PU decreased the overall crystallinity of CP. SEM analysis revealed that although all the components were uniformly dispersed in the polymer matrix, the composites had a rough and uneven surface that affected their mechanical properties. The TS of neat CP was 93.2 MPa, which decreased to 14.1 MPa for the 1:0.5 CP/PU composite and increased to 29.5 MPa for the 1:1 CP/PU composite. However, the EB of CP declined monotonically with the addition of PU. The CP/PU/curcumin composite also exhibited excellent thermal stability. The T_g_ and T_5%_ values of the composites were in the range 147.4–154.2 °C and 87.2–182.3 °C, respectively. Moreover, the addition of PU and curcumin to the CP matrix decreased the hydrophilicity of the CP/PU/curcumin composite, which in turn improved the water barrier properties and SR of the CP/PU/curcumin composite. The CP/PU/curcumin composite exhibited excellent antioxidant properties and all the composites were found to be non-cytotoxic when tested on the HaCaT cell line. Nevertheless, further research is required to better understand the mechanical properties of these composites and to establish their utility in packaging applications.

## Figures and Tables

**Table 1:** UV-transmittance, thickness, and color values of the CP/PU/curcumin composite films.

Sample	Thickness (mm)	T (%) at 600 nm	Water Vapor Permeability (×10^−9^ gm/m^2^ Pas)	Water Contact Angle (^o^)	Swelling Ratio (%)
Only CP	0.07	86.2	3.96	51.4	285
1:0.5 CP/PU	0.09	65.1	2.34	55.6	262
1:1 CP/PU	0.10	41.2	1.81	58.2	215
1:1 CP/PU with 10 wt% of curcumin	0.09	32.9	1.75	60.1	202

**Table 2:** Thermal properties, mechanical properties, WVP, and CA of the CP/PU/curcumin composite films.

Sample	TGA			DSC		Tensile Strength (MPa)	Elongation at Break (%)
	T_5%_	T_10%_	CY (%)	T_g_ (°C)	T_m_ (°C)		
Only CP	87.2	166.7	7.8	147.4	187.3	93.2	8.1
1:0.5 CP/PU	136.8	227.7	11.5	151.2	191.1	14.1	3.7
1:1 CP/PU	172.1	247.5	14.8	153.4	202.4	29.5	3.9
1:1 CP/PU with 10 wt% of curcumin	182.3	249.8	15.7	154.2	204.2	30.5	3.7

## Data Availability

Not applicable.

## Supplementary Materials

The following supporting information can be downloaded at: https://www.mdpi.com/article/10.3390/gels8040248/s1, Table S1: Cellulose pulpn (CP)/polyurethane (PU)/Curcumin composite composition.
