# Supplementary material for: Properties of Cellulose Pulp and Polyurethane Composite Films Fabricated with Curcumin by Using NMMO Ionic Liquid
Source: Gels. 2022 Apr 18;8(4):248. doi: 10.3390/gels8040248 (PMC9029826; doi:10.3390/gels8040248)
Supplement: Supplementary file 1 [file gels-08-00248-s001.zip › gels-1644414-supplementary.pdf]

Supplementary material

Table S1. Cellulose pulpn (CP)/polyurethane (PU)/Curcumin composite composition.

Sample	Cellulose Pulp (grams)	Polyurethane (grams)	Curcumin (grams)	N-methylmorpholine N-oxide (NMMO) (grams)
CP	8	0	0	92
1:0.5 CP/PU	5	2.5	0	92
1:1 CP/PU	4	4	0	92
1:1 CP/PU with 10 wt% of curcumin	4	4	0.8	92

Characterization methods

The FT-IR spectra of the prepared composites were obtained using a PerkinElmer spectrometer (Spectrum 100, USA). The XRD patterns of the composite films were obtained using an X-ray diffractometer (D/Max, Rigaku, Japan). The XRD spectra were recorded at room temperature using Cu Kα radiation as the incident X-ray source (40 kV, 200 mA) with a scanning speed of 2°/min for 2θ in the range 10–80°. Differential scanning calorimetry (DSC) was performed on the composite films using a DSC instrument (TA Instruments, USA) at a heating rate of 10 °C/min (25–300 °C) in an N_2 atmosphere. Thermogravimetric analysis (TGA) was carried out at 10 °C/min up to a temperature of 600 °C in an N_2 atmosphere using an SDT Q600 (TA Instruments, USA). The optical properties of the prepared composite films were characterized using a Shimadzu spectrophotometer (UV-1601, Japan) in the wavelength range 200–800 nm. The color properties of the composite films were measured using a tristimulus color analyzer (DP-400, Konica Minolta Inc., Japan), which was calibrated using a white porcelain reference plate. The contact angle (CA) of water on the film surface was measured using a CA analyzer (OCA 20, GmbH, Germany) having a movable stage. A drop of water (~10 μL) was placed on the film surface using a microsyringe. The water vapor permeability (WVP) of the prepared films was then determined using the ASTM E-96-95 standard method. The water vapor mass flux (WVTR) in g m^{-2} s^{-1} was determined using the slope of the steady-state (i.e., linear) portion of the weight change versus time curve. The WVP (g m^{-1} s^{-1} Pa^{-1}) of the film was determined using the following equation:

$$WVP = (WVTR \times L/\Delta p) \times 100, \qquad (1)$$

where L is the mean film thickness (m) and Δp is the gradient of partial water vapor pressure across the film. For measuring the swelling ratio (SR), the composite films were cut into smaller films of dimension 4.5 cm × 2.54 cm, following which they were immersed in distilled water at 25 °C for 24 h. Subsequently, the films were taken out of water, the excess water was removed using a blotting paper, and the films were weighed. The SR was then computed using the following equation:

$$SR\ (\%) = \frac{W_S - W_D}{W_S} \times 100,$$

where W_S and W_D are the masses of the hydrogel composites in the swollen and dry states, respectively (39). After coating with platinum, the morphology of the films was studied using an SEM (Hitachi S-4100) operated at an accelerating voltage of 10 kV. The mechanical properties of the composite films were measured using a universal testing machine (Model 3345, Instron, USA) with a crosshead speed of 300 mm/min and gauge length of 200 mm. The thickness of the composite films was determined using a digital micrometer (Mituto, Japan). The thickness of each film was measured at five different locations on the

specimen and then the average value was reported. All experiments were repeated four times. The DPPH radical scavenging potential of the composites was determined using the method discussed in (40, 41) with some modifications. Approximately 1 cm of CP and CP/PU/Curcumin composite films were added to 2 mL of methanolic DPPH (6×10^{-5} mol L^{-1}). This mixture was shaken vigorously and then kept undisturbed in a dark place for 60 min. After incubation, 200 μL of the reaction mixture (i.e., DPPH solution) was transferred to a 96-well plate, and the color intensity was measured at 517 nm using a microplate reader (Infinite M200, Tecan Group Ltd., Männedorf, Switzerland). The percentage DPPH radical scavenging activity was calculated using the following equation:

$$\text{DPPH radical scavenging activity (\%)} = \frac{\text{Absorbance of control} - \text{Absorbance of}}{\text{Absorbance of control}} \times 100.$$

The cytotoxicity potential of the synthesized composites was determined using the WST-1 assay method on HaCaT cell lines, as reported by Ravichandiran et al. (2020). HaCaT cells (1×10^{5} cells) were seeded in 24-well plates containing 500 μL of MEM and various synthesized composites (1 × 1 cm), following which the cells were incubated at 37 °C for 24 h. Subsequently, the WST-1 assay solution (50 μL/well) was added to the wells and the cells were again incubated at 37 °C for 2 h. Finally, the absorbance was measured at 450 nm using a microplate reader (Bio-Rad iMark microplate absorbance reader). All experiments were performed in triplicate. The absorbance values were expressed as a percentage of the control, which corresponds to 100% cell viability. Note that the wells without any composite films served as the control (i.e., untreated wells). Thus, the effect of CP/PU/Curcumin composites on the proliferation of HaCaT cells was expressed as the percentage of cell viability.

References

1. Balasubramanian, R.; Srinivasan, R.; Jaewoong, L. Quaternary ammonium silane-reinforced agar/polyacrylamide composites for packaging applications. *Int. J. Biol. Macromol.* **2021**, *182*, 1301–1309. https://doi.org/10.1016/j.ijbiomac.2021.05.060.
2. Srinivasan, R.; Natarajan, D.; Shivakumar, M.S. In vitro evaluation of antioxidant, antiproliferative potentials of bioactive extract-cum-rutin compound Isolated from Memecylon edule leaves and its molecular docking study. *J Biol. Active Prod. Nature.* **2016**, *6*, 43–58. https://doi.org/10.1080/22311866.2016.1173592.
3. Srinivasan, R.; Natarajan, D.; Shivakumar, M.S.; Nagamurugan, N. Isolation of Fisetin from Elaeagnus indica Serv. Bull. (Elaeagnaceae) with antioxidant and antiproliferative activity. *Free. Radicals. Antioxid.* **2016**, *6*, 145–150. https://doi.org/10.5530/fra.2016.2.3.
4. Srinivasan, R.; Aruna, A.; Manigandan, K.; Pugazhendhi, A.; Kim, M.; Shivakumar, M.S.; Natarajan, D. Phytochemical, antioxidant, antimicrobial and antiproliferative potential of Elaeagnus indica. *Biocat. Agr. Biotechnol.* **2019**, *20*, 101265. https://doi.org/10.1016/j.bcab.2019.101265.
5. Srinivasan, R.; Indhira, D.; Aruna, A.; Ashutosh, B.; Manikandan, K.; Jong, S.L.; Natarajan, D.; Myunghee, K. Functional, nutritional, antinutritional, and microbial assessment of novel fermented sugar syrup fortified with pre-mature fruits of Totapuri mango and star gooseberry. *LWT–Food. Sci. Technol.* **2021**, *136*, 110276. https://doi.org/10.1016/j.lwt.2020.110276.
6. Ravichandiran, P.; Prabakaran, D.S.; Bella, A.P.; Anna, B.C.; Maciej, M.; Krishnamoorthy, D.; Princy, M.J.; Park, B.H.; Han, M.K.; Kim, H.G.; et al. Naphthoquinone-Dopamine Linked Colorimetric and Fluorescence Chemosensor for Selective Detection of Sn^{2+} Ion in Aqueous Medium and Its Bio-Imaging Applications. *ACS Sustain. Chem. Eng.* **2020**, *8*, 10947–10958. https://doi.org/10.1021/acssuschemeng.0c03548.
